# Condition Assessment of PC Tendon Duct Filling by Elastic Wave Velocity Mapping

**DOI:** 10.1155/2014/194295

**Published:** 2014-03-06

**Authors:** Kit Fook Liu, Hwa Kian Chai, Nima Mehrabi, Kobayashi Yoshikazu, Tomoki Shiotani

**Affiliations:** ^1^Department of Civil Engineering, University of Malaya, Kuala Lumpur, Malaysia; ^2^Department of Civil Engineering, Nihon University, Tokyo, Japan; ^3^Graduate School of Engineering, Kyoto University, Kyoto, Japan

## Abstract

Imaging techniques are high in demand for modern nondestructive evaluation of large-scale concrete structures. The travel-time tomography (TTT) technique, which is based on the principle of mapping the change of propagation velocity of transient elastic waves in a measured object, has found increasing application for assessing in situ concrete structures. The primary aim of this technique is to detect defects that exist in a structure. The TTT technique can offer an effective means for assessing tendon duct filling of prestressed concrete (PC) elements. This study is aimed at clarifying some of the issues pertaining to the reliability of the technique for this purpose, such as sensor arrangement, model, meshing, type of tendon sheath, thickness of sheath, and material type as well as the scale of inhomogeneity. The work involved 2D simulations of wave motions, signal processing to extract travel time of waves, and tomography reconstruction computation for velocity mapping of defect in tendon duct.

## 1. Introduction

Prestressed concrete (PC) is recognized as a vital technology to overcome concrete's natural weakness in tension especially for bridge structure. However, improper grouting of tendon duct can lead to deterioration inside the bridge structure which includes cracking and corrosion of tendons. The corrosion in tendon duct leads to lack of cross-sectional area and increases stress to the other tendons, finally results in bridge collapse [1]. To avoid collapse of a deteriorated structure or other disaster, proper preventive maintenance has to be carried out. Nondestructive testing has to be carried out to detect which part of bridge structure is defected so that repair work can be carried out rapidly and cost efficiently. Besides, it is encouraged to conduct inspection on a regular basis so that necessary considerations and effective repair action can be implemented. There are various nondestructive techniques (NDT) that have been practiced by engineers in detecting defects and evaluating the integrity of structures depending on the physical conditions of concrete structures. NDT is a group of techniques that evaluate the properties of material, component, or system without impairing its future usefulness. The unique advantages of employing NDT include time and cost saving, flexibility in operation, and simple implementation. The results are useful in providing warning or indication towards imminent failure.

NDT is a very demanding profession for assessment of concrete structures because destructive methods such as coring and drilling will leave permanent damage and are costly. Also, defects that are left by destructive methods potentially become focal points for deterioration. Several NDT methods have been used so far for assessment of PC, which include impact-echo, radar technique, radioactive, and impulse thermography. The impact-echo method employs elastic wave to propagate in concrete structure and elastic wave will reflect on concrete surface when it meets with internal defects. This method is applied to pre- and posttensioned concrete structure to determine the location of the defect. For impact-echo method, it is essential to ensure that impact frequency is sufficiently high to identify the defect. However this technique is not always accurate because many peak frequencies are observed in the frequency spectra due to reflection, diffraction, and so forth. To circumvent this, a new procedure was developed by applying an imaging procedure to the impact-echo data, as stack imaging of spectral amplitudes based on impact-echo (SIBIE) [2, 3]. Alver and Ohtsu had conducted lab tests regarding SIBIE procedure which is found to be efficient to identify specimens containing grouted and ungrouted ducts [4]. The state-of-the art ultrasonic tomography (MIRA) is a device manufactured by Acoustic Control Systems, which contains four rows of sensors in which each row contains ten transducers. The impact-echo needs one receiver sensor for one measurement, while the MIRA used the same concept as impact-echo. MIRA is an ultrasonic tomography device that was developed to determine the depth of concrete pavement and reinforcement location as well as detection of flaws in concrete pavement or PC. However, it is suggested to use the MIRA with combination of other NDT techniques because of the inability of the device to determine the exact location of flaws at big areas by short time [5]. On the other hand, radar technique such as the electromagnetic (EM) wave method can be rapid to locate and image defects inside of concrete structures effectively. However, radar techniques are found to have imaging limitations such as diffraction which might affect the visualization, lack of exact inversion algorithms, loss of polarization information due to scalar inversion, and high attenuation of EM waves in moisture [6]. There are experiments which were conducted by Langenberg et al. to assess the condition of PC tendon duct by using EM wave; generally the EM waves face the problem of being shielded by the steel grid and the tendon duct itself [7]. Radioactive methods such as X-ray can provide high resolution result of tendon duct images as X-ray is nondiffracting and possesses high penetration capability. But there are limitations for X-ray methods such as high operating costs and safety issues due to risk of radiation. Impulse thermography can be applied to detect subsurface defects of tendon duct, either by heat hydration of the grout, infrared array, or electric resistance. Defects can be identified when there are areas of different temperatures as a result of transient heat transfer. This method is effective for penetration up to 10 cm, and identification is good only at an early stage of concrete hardening [8]. But, Clark et al. found that weather and surface conditions can affect the accuracy of imaging by impulse thermography [9].

In this study, the NDT method in focus is the travel-time tomography (TTT) reconstruction technique, which provides assessment through variation of travel time of elastic waves from a source to multiple receivers located at different locations of the target structure. The travel time, or inversely the velocity of elastic wave, depends on the medium of propagation. The governing properties that influence the behaviour of propagation include density and acoustic impedance, as well as homogeneity of the material [10–14]. According to Vergara et al. [15], wave propagation is also affected by inhomogeneity in the forms of porosity and aggregates in concrete. For assessment purposes, there are established empirical correlations between wave velocity and concrete quality [16, 17]. Commonly, velocities higher than 3500 ms^−1^ indicate sound concrete, while those lower than 3500 ms^−1^ are common in normal concrete with questionable integrity [18]. The delay or low velocity of observed data can always be associated with the presence of an anomaly or defect, in the form of cracking or voiding.

A significant merit for TTT method in civil structures maintenance industry is that the tomography results can be kept as an appraisal record for new construction and as a source of reference to evaluate the status of deterioration or damage during the service period [18]. In order to increase the reliability of TTT assessment and to confirm proper instrumentation and measurement configuration, it is often necessary to conduct wave motion simulation because the size, material characteristics, and geometry of concrete structures are varied from one to another. Simulation work is necessary to obtain important information such as optimum conditions for sensor arrangement and frequency of the wave. Likewise, through simulation, decisions can be made in terms of the required number of sensors, required assessment sides of the structure, and the size of meshing for satisfactory visualization to ensure cost-effectiveness of measurement. Beside velocity variation, attenuation of elastic waves is considered to be a more sensitive variation against homogeneity. Nevertheless, due to significant attenuation of elastic waves through cementitious medium especially with defects, the velocity and attenuation tomography might not be suitable for utilizing on large scale structure because of the loss of signal amplitude even in concrete free of defect. Attenuation is also considered very dependent on the coupling and surface conditions, while velocity is not as much [19].

### 1.1. Tomography by Travel Time of Elastic Waves

**Figure 1:**
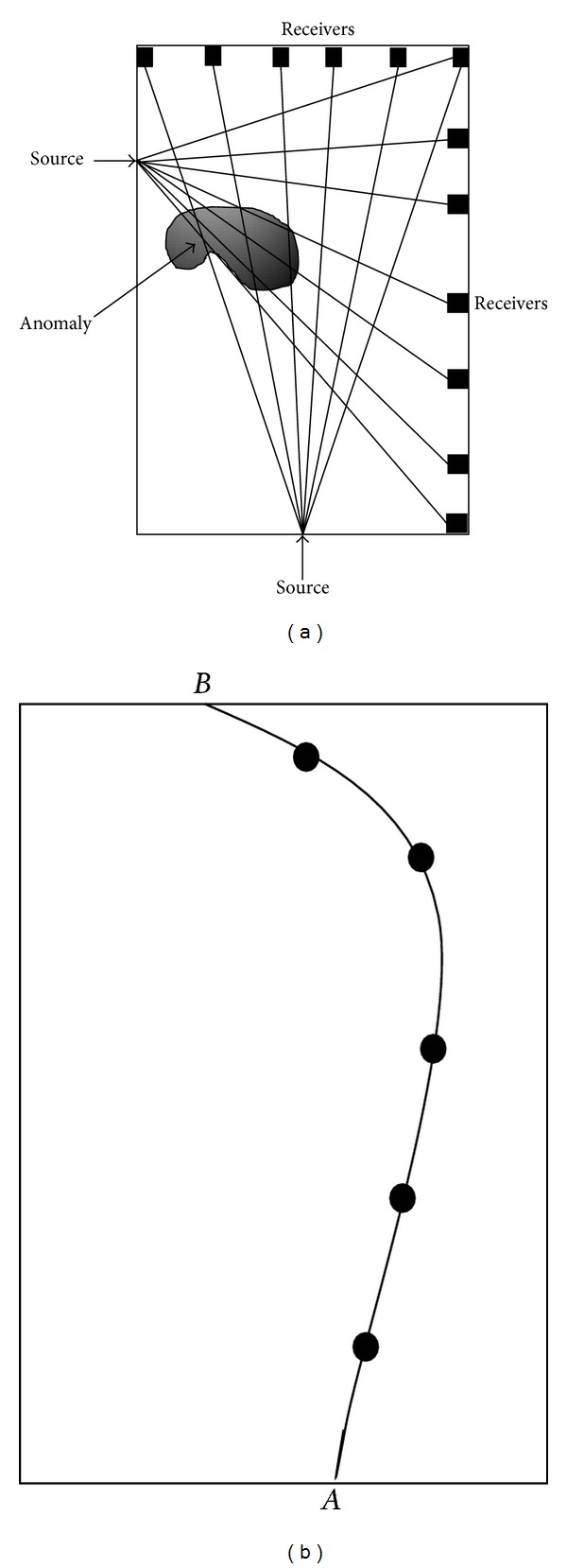
(a) Schematic illustration of anomaly projections with ray trace from source nodal to multiple receivers [1]; (b) an example of one-ray traces from point *A* to point *B* based on illustration (a).

The TTT is a type of transmission tomography technique that employs elastic waves to propagate on the target structure medium from one source to multiple receivers as shown in Figure 1(a). The inversion of travel-time data enables for tomographic imaging of the velocity distribution within the sampled structure. It is known that elastic waves propagate at varying velocities in different materials and are highly dependable upon the physical properties of the medium, such as elastic modulus and acoustic impedance of laying materials. This is because when there is heterogeneity in a medium or existence of voids, the elastic waves will experience scattering, reflection, and diffraction; in such a loss of energy the properties of waves such as frequencies and amplitude may change [20]. This method allows better identification of anomalous regions by performing an inversion of boundary measurements to determine the physical properties within the body of a structure. The visualization of tomogram should be highly presentable and easy to be understood not only by NDT engineers but also by people without the relevant technical background, such as owners of structures. The following section describes inversion of travel-time data which has been practiced by this experiment.

Based on the ray theory, the travel time *t*, for the wave to travel from source *A* to receiver *B* (as in Figure 1) is given by the path integral [18]:

(1) Travel  time, t=∫AB1v  dl=∫ABs dl,

where the integral follows the ray path from point *A* to point *B* with *v* as velocity, *s* is the wave slowness (also known as reciprocal of velocity), and *dl* is the element length. Based on the series expansion technique, *s*(*x*, *z*) as a set of discrete elements or pixels, each with a uniform slowness, *s*_*j*_ (*j* = 1, *M*, where *M* is the number of pixels). Then the integral corresponding to travel time, *t*_*i*_ (*i* = 1, *N*, where *N* is the number of observations) becomes a summation:

(2) ti=  ∑j=1Mpjdij (i=1,…,N),

where *d*_*ij*_ is the distance travelled by ray *i* in pixel  *j*. For the whole set of rays, the travel time equation above can be represented in matrix form as

(3) $$\begin{array}{c} T=DP, \end{array}$$

where *T* and *P* are column vectors of length *N* and *M*, respectively, and *D* is an *N* by *M* rectangular matrix. Mesh velocity of tomogram are results from solving the slowness vector, *P* from observation travel time matrix, *T* and path length, *D*. To solve the slowness vector *P*, it is required to measure travel time, *T*, and transpose matrix D*:

(4) $$\begin{array}{c} P=D^{*T}T, \end{array}$$

where the matrix D*  is obtained by dividing each row of *D*, corresponding to a particular ray path, by the square of the path length. Thus,

(5) $$\begin{array}{c} P_{j}=\overset{N}{\underset{i=1}{\sum }}\frac{D_{ij\;}T_{i}}{D_{i}^{2}}\;\left(j=1,\ldots ,M\right). \end{array}$$

The Equation (5) can be expressed as matrix form in Equation (6) and it can be solved until it satisfies the inconsistent data as closely as possible.

(6) $$\begin{array}{c} P={\left(D^{T}D\right)}^{-1}D^{T}T. \end{array}$$

Since this group of matrix involves a high amount of order and value, the simultaneous iterative reconstruction techniques (SIRT) program is developed to reconstruct the velocity distribution across the structure.

In this research, wave motion simulation was used to generate data for the TTT method in assessing the filling of the PC tendon duct. The objective of this study is to investigate the effect of sensors arrangement, mesh size and number of elements, material of tendon sheath, and thickness of tendon sheath on the reliability of TTT measurement. Discussion is also extended to analyse the TTT results for quantitative evaluation of filling.

## 2. Methodology

### 2.1. Numerical Simulations

The analytical work was carried out by two-dimensional (2D) numerical simulations of wave motions. The numerical simulations were conducted with commercially available software named Wave2000 Plus developed by CyberLogic, Inc., in order to produce the raw data (travel time). The simulation results were further processed and the data were used as the input data for tomography reconstruction computation to generate tomograms that indicate the velocity distribution of the interior of the measured target. The fundamental equation of two-dimensional propagation of stress waves in a perfectly elastic medium, by ignoring viscous losses, is as follows:

(7) $$\begin{array}{c} p\frac{\partial^{2}u}{\partial t^{2}}=\mu \nabla^{2}u+\left(\lambda +\mu \right)\cdot u, \end{array}$$

where *u* = *u*(*x*, *y*, *t*) is the time-varying displacement vector, *p* is the material density, *λ* and *μ* are the first and second lame constants, and *t* is the travel time. Equation (7) can be solved by using the finite difference method in the plane strain case. The software performs computation to solve the equation at discrete points with respect to the boundary conditions of the model, which include the input source that has predefined time-dependent displacements at a given location and a set of initial conditions. The above equation is applicable for wave propagation to solve any heterogeneous geometry, while the continuity conditions for stress and strains must be satisfied on the interfaces.

### 2.2. Material Modeling

**Figure 2:**
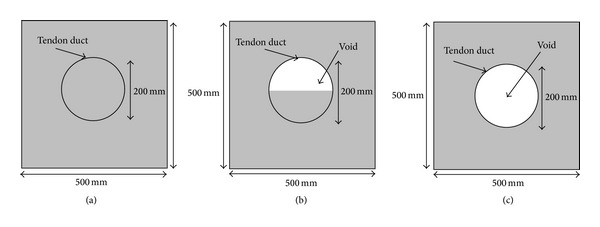
Schematic diagrams of simulation models showing filling of duct at (a) 100%, (b) 50%, and (c) 0%.

There are basically three types of models as illustrated in Figure 2, which were tested with TTT reconstruction technique. The concrete model was a 500 mm × 500 mm cross section with a 250 mm diameter tendon duct at the center. There are two types of material for tendon sheath under investigation: aluminium, and polyethylene. The density of concrete, aluminium and polyethylene has been selected as 2400 kgm^−3^, 2700 kgm^−3^, and 1050 kgm^−3^, respectively. Based on the density of materials, it will give wave velocity of 4000 ms^−1^, 6400 ms^−1^, and 2400 ms^−1^ for concrete, aluminium, and polyethylene, respectively. Besides, two different thickness values for tendon sheath were used in the simulation, namely, 1 mm and 10 mm. The purpose of selecting 1 mm and 10 mm for the thickness of the tendon duct is to study the effect of tendon duct thickness on tomogram.

**Figure 3:**
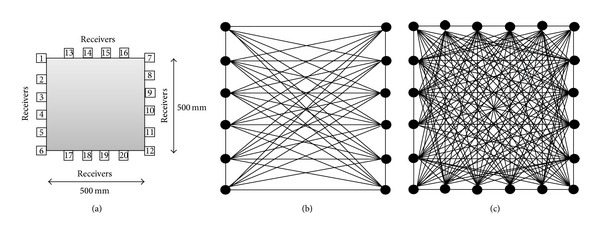
(a) Arrangement of sensors on 500 mm × 500 mm concrete specimen; (b) ray-path coverage with 2-side transducers; (c) ray-path coverage with 4 sides transducers (complete coverage).

Twenty sensors with a size of 20 mm each were located as shown in Figure 3. The distance between each two sensors was 100 mm. The source was configured as a single cycle of Sine Gaussian pulses at 50 kHz frequency. For the cases of 2-side transducers coverage, when one of the sensors (sensor 1) was set as the source, all other sensors at sides different from the source (sensors 1–12) would be set as receivers to record the transmitted wave. The ray-path coverage associated with 2-side transducers coverage is shown in Figure 3(b). For the cases of 4-side transducers coverage (complete coverage), when one of the sensors (sensor 1) was set as the source, all other sensors at sides different from the source (sensors 1–20) would be set as receivers to record the transmitted wave. The ray-path coverage associated with 4-side transducers coverage is shown in Figure 3(c). Travel time was extracted through identification of the onset of the arriving wave signal at the receivers. The source is shifted to a subsequent sensor position in a specific order and the transmission, receiving process was continued. This would eventually give a set of travel-time data to be used as observed data for TTT computation. Based on the input mechanical properties of concrete, in a sound medium, the wave velocity will be higher than 4000 ms^−1^, while in the velocity range of 3900–4000 ms^−1^ medium is considered weak. In the region of the void, the travel velocity of the wave falls lower than 3900 ms^−1^.

## 3. Results

The visualization of concrete interior by tomography reconstruction technique is mainly affected by three factors, namely,

1. number and arrangement of sensors,
2. size of element or number of cells,
3. type or thickness of PC tendon duct.

### 3.1. Effect of Number and Arrangement of Sensors

#### 3.1.1. Effect of 1 mm Thick Polyethylene Tendon Sheath

**Figure 4:**
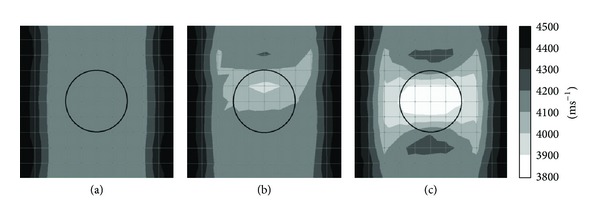
Tomograms of model with a total number of 12 sensors attached to left and right sides: (a) 0% void; (b) 50% void; (c) 100% void.

**Figure 5:**
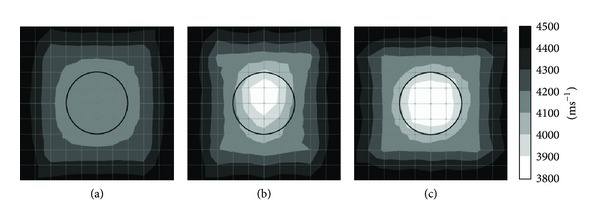
Tomograms of model with a total number of 20 sensors attached to four sides: (a) 0% void; (b) 50% void; and (c) 100% void.

Figures 4 and 5 show tomograms for a concrete specimen with the interior containing 1 mm thick polyethylene tendon duct for the three filling conditions of the sheath simulated with 100 elements. The black colour at the right and left sides of the tomogram illustrated in Figure 4(a) indicates velocity range higher than 4500 ms^−1^. The middle part of the tomogram shows velocity in the range of 4100–4200 ms^−1^, which is less than the left and right sides of the tomogram. The reason is lack of sensor attachment at the top and bottom of the specimen for recording the waveform. Since the TTT faced insufficiency of data on top and bottom parts of concrete specimen, it assumed an average data for the region near the top and bottom of concrete specimen. On the contrary, Figure 5(a), in which the visualization contains 20 sensors attached to four sides, shows relatively higher velocity of elastic waves than that in Figure 3(a).

The tomogram shown in Figure 4(b) contains 50% void inside 1 mm thickness polyethylene tendon duct. The result of visualization succeeded to spot a drop of velocity to less than 4000 ms^−1^ on the location of the void. Meanwhile, the tomogram in Figure 5(b) which is of a specimen with 20 sensors attached to four sides is clearer to spot the velocity of wave propagation lower than 3900 ms^−1^ on the location of the void.

Comparing the tomogram of Figure 4(c) showing the white colour (elastic wave velocity less than 3900 ms^−1^) region on the center of the tendon duct with an ellipse shape to the one in Figure 5(c) which contains 20 sensors attached to four sides, the tomogram of Figure 5(c) succeeds to show the correct shape of the empty duct with a velocity lower than 3900 ms^−1^.

#### 3.1.2. Effect of 1 mm Thick Aluminium Tendon Sheath

**Figure 6:**
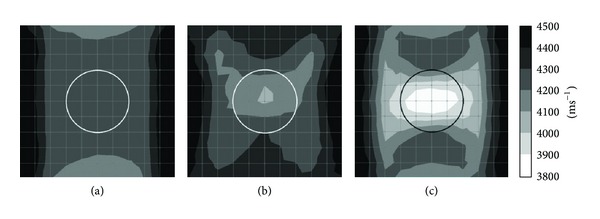
Tomograms of model with a total number of 12 sensors attached to left and right side: (a) 0% void; (b) 50% void; (c) 100% void.

**Figure 7:**
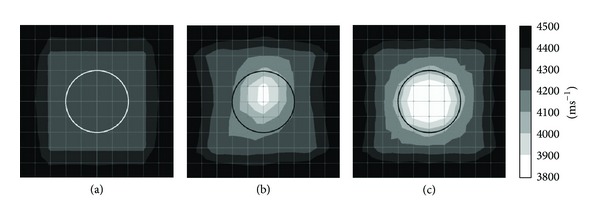
Tomograms of model with a total number of 20 sensors attached to four sides: (a) 0% void; (b) 50% void; and (c) 100% void.

Figures 6 and 7 show tomograms for concrete with the interior containing 1 mm thick aluminium tendon duct simulated with 100 elements. The tomogram in Figure 6(a) indicates that the velocity of wave falls into region of 4100–4200 ms^−1^ at the top and bottom parts coloured in grey. However, left side and right side of concrete model show velocity higher than 4200 ms^−1^. While the tomogram as given in Figure 7(a) shows more consistency in visualization if compared to tomogram shown in Figure 6(a).

Tomograms illustrated in Figures 6(b) and 7(b) are related to the model with 50% void inside 1 mm thick aluminium tendon duct. The tomogram in Figure 6(b) was successful to spot the 50% void region with wave propagation velocities between 3900 and 4000 ms^−1^. Meanwhile, the tomogram given in Figure 7(b) was successful to spot a void with wave velocity lower than 3900 ms^−1^ on the location of 50% void although it seems to be smaller than it is supposed to be. The wave velocity increases from the center of a concrete specimen to the four edges. From cases of Figures 6(b) and 7(b), we can clarify that the TTT is able to spot 50% void inside of 200 mm diameter aluminium tendon duct if there are 20 sensors attached to four sides of the 500 mm × 500 mm concrete specimen.

The tomograms given in Figures 6(c) and 7(c) both are related to the model with 100% void in the center of tendon duct. The one in Figure 6(c) has only 12 sensors attached to the left and right sides of a concrete specimen which is sufficient to spot the void by indicating velocity lower than 3900 ms^−1^. Although Figure 6(c) was unable to spot the void with the same size as the tendon duct, it indicates that the wave velocity in the tendon duct is lower than 4000 ms^−1^. The tomogram in Figure 7(c) can spot the void with velocity lower than 3900 ms^−1^ which is almost the same size as the aluminium tendon duct.

### 3.2. Effect of Size and Number of Elements

#### 3.2.1. Effect of Number and Size of Elements by 10 mm Thick Polyethylene Tendon Sheath

**Figure 8:**
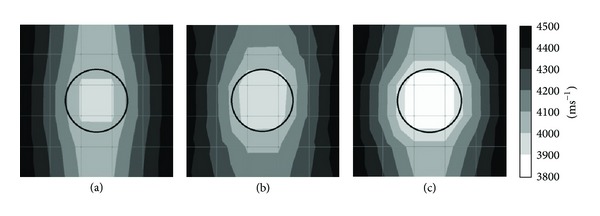
Tomograms simulated by 25 elements: (a) 0% void; (b) 50% void; and (c) 100% void.

**Figure 9:**
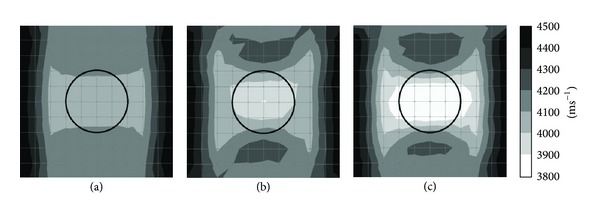
Tomograms simulated by 100 elements: (a) 0% void; (b) 50% void; and (c) 100% void.

*(1) Effect of 10 mm Thick Polyethylene Tendon Sheath with 2-Side* *Sensor Attachment*. Figures 8 and 9 show the tomograms of a concrete specimen containing 10 mm polyethylene tendon duct with 12 sensors attached to both sides. Tomogram in Figure 8(a) is simulated into 25 elements or 100 mm × 100 mm element size while tomogram in Figure 9(a) is simulated with 100 elements or 50 mm element size; the inside of the tendon duct of model in Figure 8(a) shows a rectangular shape region with velocity in the range of 3900–4000 ms^−1^. The tomogram in Figure 9(a) shows the center of specimen with wave velocity range of 3900–4000 ms^−1^. In both cases, the top and bottom of tomogram show slower wave velocity due to the insufficient data caused by lack of sensor arrangement at the top and bottom parts of the specimen.

The tomogram as given in Figure 8(b) is simulated with 25 elements but it failed to visualize the void. However, it detected the inside of 10 mm polyethylene tendon duct with wave velocity in the range of 3900–4000 ms^−1^. Meanwhile, tomogram in Figure 9(b) shows an ellipse with velocity 3900–4000 ms^−1^ in the center of the tendon duct. There are also recognitions of two increasing velocities (range of 4200–4300 ms^−1^) in the area between the top and bottom of the tendon duct with ellipse shape.

Tomogram shown in Figure 8(c) is simulated with 25 elements and it succeeded to identify the void with velocity lower than 3900 ms^−1^ with the same size as tendon duct which is 200 mm in diameter. Figure 9(c) shows a tomogram simulated by 100 elements and it succeeded to detect the void with wave velocity lower than 3900 ms^−1^ but it is in ellipse shape. The visualization of tomogram in Figure 9(c) is not satisfying since it is shown that void is tabulated to the outside of the tendon duct. Also, the visualization of tomogram given in Figure 9(c) is more confusing than that of Figure 8(c).

By referring to the visualization of tomograms of Figures 8 and 9, for the model of concrete with 10 mm thick polyethylene tendon duct, if 12 sensors are attached to both sides, it is suitable to simulate the model by 25 elements or 100 mm element size and it is unnecessary to utilize 100 elements or 50 mm element size since the result will not improve. Moreover, the tomogram with 100 degrees of freedom can cause confusion if compared to tomogram with 25 degrees of freedom.

**Figure 10:**
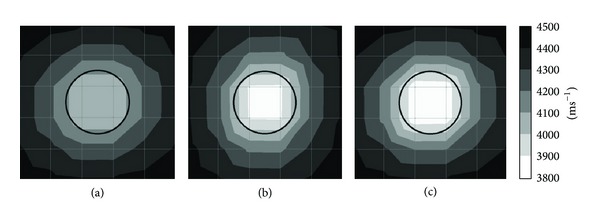
Tomograms simulated by 25 elements: (a) 0% void; (b) 50% void; and (c) 100% void.

**Figure 11:**
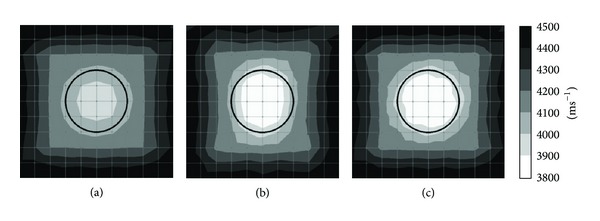
Tomograms simulated by 100 elements: (a) 0% void; (b) 50% void; and (c) 100% void.

*(2) Effect of 10 mm Thick Polyethylene Tendon Sheath with 4-Side* *Sensor Attachment.* Figures 10 and 11 are related to the tomograms of a concrete specimen with 20 sensors attached to 4 sides. The interior of concrete specimen is 10 mm thick polyethylene tendon sheath.  Figure 10 presents the tomogram which is simulated by 25 elements while Figure 10 presents the tomogram simulated by 100 elements. Figures 10(a) and 11(a) both have the same physical geometry and the results of visualization are acceptable. The models of Figures 10(b) and 11(b) have 50% voids inside of the tendon duct; both tomograms did spot the void with wave velocity lower than 3900 ms^−1^. The visualization of tomogram Figure 10(b) seems to be better than the visualization of Figure 11(b). Nevertheless, the model shown in Figure 11(b) is simulated by 100 elements; it shows that the void is bigger than it was supposed to be. The tomograms as given in Figures 10(c) and 11(c) have 100% void inside tendon duct and both succeed to identify the void with wave velocity lower than 3900 ms^−1^. The tomogram in Figure 10(c) shows void with square shape while tomogram of Figure 11(c) shows void with a circular shape.

#### 3.2.2. Effect of Number and Size of Elements by 1 mm Thick Aluminium Tendon Duct

**Figure 12:**
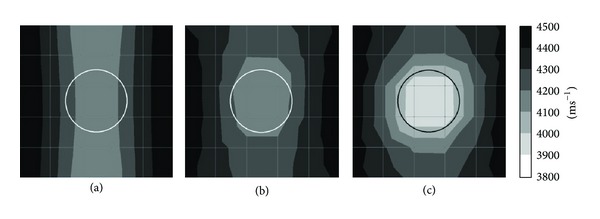
Tomograms simulated by 25 elements: (a) 0% void; (b) 50% void; and (c) 100% void.

*(1) Effect of 1 mm Thick Aluminium Tendon Duct with 2-Side Sensor Attachment.* Figures 6 and 12 are the tomograms of a concrete specimen containing 1 mm aluminium tendon duct with 12 sensors attached to both sides. Tomogram of Figure 6 has been simulated into 25 elements with a size of 100 mm × 100 mm, while the one in Figure 12 is simulated by 50 mm × 50 mm. The top, middle, and bottom of tomogram in Figure 11(a) show velocity with range of 4000–4100 ms^−1^ while the rest meshes show higher velocity. The tomogram shown in Figure 6(a) shows the drop of velocity from 4100 ms^−1^ to 4000 ms^−1^ on the top and bottom parts of model.

Both of the models shown in Figures 6(b) and 12(b) have 50% void inside 10 mm thick aluminium tendon duct. Although both of the models failed to detect void with velocity lower than 3900 ms^−1^, tomogram in Figure 6(b) shows a spot with velocity in the range of 3900–4000 ms^−1^.

In case of Figures 6(c) and 12(c), the model contains 100% void inside 10 mm thick aluminium tendon duct. Figure 12(c) interprets that the interior of the tendon duct with velocity in the range of 3900–4000 ms^−1^. The model of tomogram in Figure 6(c) can visualize the void at the center of concrete interior with velocity lower than 3900 ms^−1^. The shape of void in Figure 6(c) is in horizontal major axis elliptical shape yet it is smaller than the size it was supposed to be. Even though models that are simulated with 100 elements did spot the 100% void inside the tendon duct with velocity lower than 3900 ms^−1^, the visualization of models simulated with 25 elements is less confusing.

**Figure 13:**
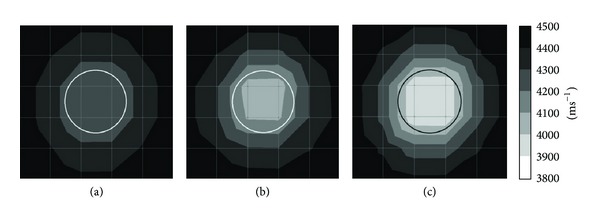
25 elements: (a) 0% void; (b) 50% void; and (c) 100% void.

*(2) Effect of 1 mm Thick Aluminium Tendon Sheath with 4-Side Sensor Attachment.* Figures 7 and 13 are the tomograms of concrete model containing 1 mm aluminium tendon duct with 20 sensors attached to four sides. The former Figure 7 was simulated into 50 mm × 50 mm element's size and Figure 13 into 100 mm × 100 mm size. Models of tomogram in Figures 13(a) and 7(a) both contain no void inside 1 mm thick aluminium tendon duct thus the center of a concrete specimen shows the wave velocity in the range of 4200 ms^−1^ to 4300 ms^−1^.

Models of tomogram in Figures 13(b) and 7(b)both contain 50% of void inside 1 mm thick aluminium tendon duct. Tomogram in Figure 13(b) shows the location of 50% void with velocity in the range of 4000–4100 ms^−1^ while tomogram in Figure 7(b) spots the void with velocity lesser than 3900 ms^−1^, although it is smaller than it is supposed to be.

Models of tomogram in Figures 13(c) and 7(c) both contain 100% of void inside 1 mm thick aluminium tendon duct. Although the model of tomogram in Figure 13(c) was unable to detect the void with velocity lesser than 3900 ms^−1^, it did show the fall of velocity into the range of 3900–4000 ms^−1^ inside the tendon duct. On the other hand, the tomogram shown in Figure 7(c) can visualize the void with velocity lesser than 3900 ms^−1^ inside the tendon duct.

### 3.3. Effect of Type and Thickness of Prestressed Concrete's Tendon Duct

#### 3.3.1. Effect of Polyethylene Tendon Sheath

Figures 5 and 11 both are tomograms of concrete model containing 100 elements with 20 sensors attached to four sides. Tomogram given in Figure 5(a) is related to the model with 1 mm thick polyethylene tendon sheath on the center of the concrete. Generally all of the tomogram elements show velocity higher than 4100 ms^−1^. While tomogram of Figure 11(a) is a tomogram of a concrete specimen which contains 10 mm polyethylene tendon duct. The tomogram in Figure 10(a) has one circle with elastic wave velocity in the range of 4000–4100 ms^−1^ at the center of the tendon duct.

Tomogram given in Figure 5(b) shows that there are voids with velocity lower than 3900 ms^−1^ on the location of 50% void inside the tendon duct. While tomogram in Figure 11(b) shows that the void (velocity lower than 3900 ms^−1^) is almost the same size as tendon duct and it is bigger than 50% of tendon duct.

Tomograms in Figures 5(c) and 11(c) both have 20 sensors attached to four sides, 100 elements, and 100% of void inside the tendon sheath. Tomogram in Figure 5(c) has 1 mm thick of polyethylene tendon sheath whereas tomogram in Figure 11(c) has 10 mm thick of polyethylene tendon sheath. There is not much difference between tomogram in Figure 5(c) and tomogram in Figure 11(c); both tomograms succeed to spot the 100% void with wave propagation velocity lower than 3900 ms^−1^ and the size of the void is same size as the tendon duct.

In short, the visualization of tomogram containing 10 mm thick polyethylene tendon duct shows relatively higher elastic wave velocity than 1 mm thick polyethylene.

#### 3.3.2. Effect of Aluminium Tendon Duct

**Figure 14:**
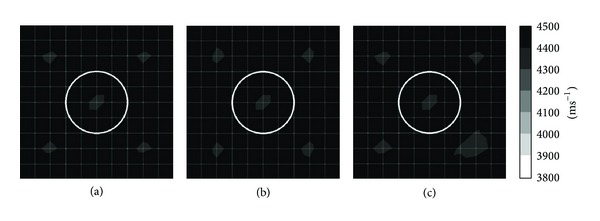
10 mm thick aluminium duct: (a) 0% void; (b) 50% void; and (c) 100% void.

Figures 7 and 14 are the tomograms of concrete model containing 20 sensors attached to four sides and simulated into 100 degrees of freedom. Tomograms in Figures 7(a), 7(b), and 7(c) have 1 mm thick aluminium tendon duct with 200 mm diameter as shown with black colour in the figure. Tomogram in Figure 7(a) relates to the model with 0% void inside the aluminium tendon duct, tomogram in Figure 7(b) relates to the model with 50% void inside the aluminium tendon duct, and tomogram in Figure 7(c) has 100% void inside the aluminium tendon duct. The visualization of tomograms in Figure 7 is acceptable. However, the other three tomograms which are tomogram in Figure 14(a), tomogram in Figure 14(b), and tomogram in Figure 14(c) have 10 mm thick aluminium tendon duct with 200 mm diameter as shown in white colour in three figures. It can be seen that beside tomogram in Figure 14(a), the visualization of tomograms in Figures 14(b) and 14(c) were unable to distinguish the size of void.

## 4. Discussion

Throughout all the tomograms in this experiment, it is found that the TTT is not applicable to detect void for 500 mm square concrete model that contains 10 mm thick aluminium tendon duct. This was mainly due to the reduced sensitivity by changing the increased aluminium thickness, which resulted in change of wave propagation behavior. The following sections will discuss the influencing factors that affect the sensitivity of tomography results with regard to different sheath material type and thickness.

### 4.1. Acoustic Impedance

Propagation of waves through a material is influenced by the sound pressure. This is because molecules or atoms in a solid material are bound elastically to each other; the excess pressure results in a wave propagating through the solid. The acoustic impedance is a physical property of material to measure how far the influence of wave propagates through the material. Different materials have different values of acoustic impedance.

Theoretically, when waves spread from a medium with higher acoustic impedance to a medium with lower acoustic impedance, the incident waves will be diffracted and the velocity also will be lowered. Mathematically,

(8) $$\begin{array}{c} Z\;=\;p\;\times \;c, \end{array}$$

where *Z* is acoustic impedance, *p* is density of material, and *c* is the velocity of wave propagates.

So, the acoustic impedance, *Z*, for aluminium:

(9) $$\begin{array}{c} Z=2700\;{\text{kgm}}^{-3}\times 6419.88\;{\text{ms}}^{-1}=17,333,676\;{\text{kgm}}^{-2}{\text{s}}^{-1}. \end{array}$$

The acoustic impedance, *Z*, for polyethylene:

(10) $$\begin{array}{c} Z=1050\;{\text{kgm}}^{-3}\times 2400.2\;{\text{ms}}^{-1}=2,520,210\;{\text{kgm}}^{-2}{\text{s}}^{-1}. \end{array}$$

The acoustic impedance, *Z*, for concrete:

(11) $$\begin{array}{c} Z=2400\;{\text{kgm}}^{-3}\times 4082.48\;{\text{ms}}^{-1}=9,797,952\;{\text{kgm}}^{-2}{\text{s}}^{-1}. \end{array}$$

Therefore,: aluminium > concrete > polyethylene > void.

Theoretically, acoustic impedance of aluminium is double that of concrete and seven times higher than polyethylene. Therefore the wave propagates in aluminium medium faster than concrete and polyethylene. Since the speed of waves travelling in aluminium medium is higher than in the concrete, it can mask the actual velocity to travel in the region of the void. Therefore, the result of visualization for the aluminium with a thickness of 10 mm is rather inaccurate and low in resolution. But once the thickness of aluminium sheath has been reduced to 1 mm, the visualization of result became satisfying and helped to detect the defect with good contrast. For the polyethylene sheath, the acoustic impedance is lower than the concrete so the visualization is more accurate. Since the wave has been diffracted once it penetrated into polyethylene and void, thus the velocity has been reduced dramatically.

### 4.2. Refraction

Refraction is a phenomenon in which the wave velocity and direction change when it enters the other medium. According to Snell's law, for a wave with a single frequency that transmit from given pair of media, the ratio of the sines of the angle of incidence, *θ*_1_ and refraction, *θ*_2_ is equivalent to the ratio of phase velocities (*v*_1_/*v*_2_) in the two media, or equivalent to the reciprocal ratio of the indices of refraction  (*n*_2_/*n*_1_):

(12) $$\begin{array}{c} \frac{\sin\theta_{1}}{\sin\theta_{2}}=\frac{v_{1}}{v_{2}}=\frac{n_{2}}{n_{1}}. \end{array}$$

Therefore, the refraction of angle from one medium to the other medium can be calculated as

(13) $$\begin{array}{c} \theta_{2}=\sin^{-1}\left(\sin\theta_{1}\times \frac{n_{1}}{n_{2}}\right). \end{array}$$

The velocity of wave propagation after transverse through other medium can be calculated as

(14) $$\begin{array}{c} v_{2}=v_{1}\cdot \;\frac{\sin\theta_{2}}{\sin\theta_{1}}=v_{1}\cdot \frac{n_{2}}{n_{1}}, \end{array}$$

where

 
*θ*_1_ = angle of incidence,
 
*θ*_2_ = angle of refraction,
 
*n*_1_ = refraction indices of incidence medium,
 
*n*_2_ = refraction indices of refraction medium.

Based on the studies by Sato et al. [21], the refractive index of concrete showed relatively little change; the value a week after concreting was 2.79, while that obtained fourteen months after concreting was 2.55. In this case, it is assumed that the refractive index is 2.55. Generally, the refractive indices of aluminium, polyethylene, and air are 1.44, 1.55, and 1.00 relatively.

**Figure 15:**
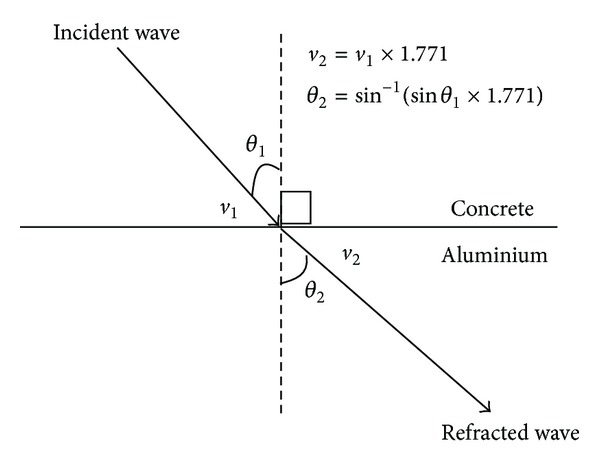
Refraction of wave from concrete medium to aluminium medium.

**Figure 16:**
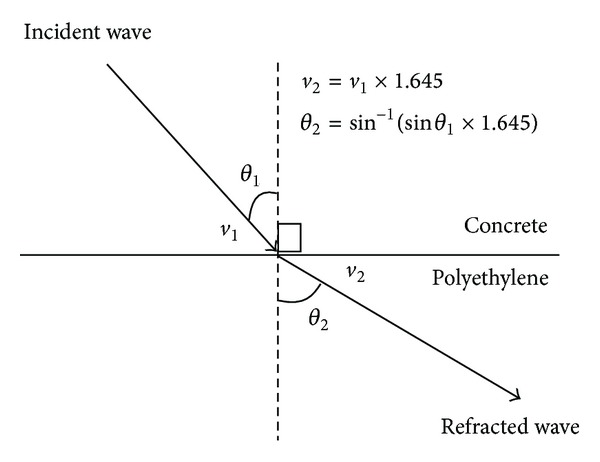
Refraction of wave from concrete medium to polyethylene medium.

Figure 15 is an illustration of ray propagation from concrete to aluminium having a refraction factor (*n*_2_/*n*_1_) of 1.771. On the hand, Figure 16 refers to wave propagating from concrete to polyethylene, the refraction factor (*n*_2_/*n*_1_) is 1.645.It shows that the refraction factor for concrete-aluminium is higher than that of concrete-polyethylene. This explains why the wave velocities of concrete model containing 10 mm aluminium thick tendon duct are relatively fast at more than 4500 ms^−1^, resulting in that the void cannot be properly visualized due to the masking effect.

### 4.3. Young's Modulus and *p*-Wave Velocity of Material

The mechanical properties such as young's modulus and *p*-wave velocity of concrete and tendon duct have a big influence on speed of wave propagation. Velocity of *p*-waves in homogeneous, semi-infinite, elastic solids is defined as

(15) $$\begin{array}{c} C_{p}=\;\sqrt{\frac{E\left(1-\sigma \right)}{\rho \left(1+\sigma \right)\left(1-2\sigma \right)},} \end{array}$$

where *C*_*p*_ is *p*-wave velocity, *E* is Yong's modulus of material, *ρ* is the density of material, and *σ* is the Poisson's ratio of material. Commonly the Poisson's ratio of aluminium and polyethylene is 0.34 which has been used in simulations. Also, the Young's modulus of aluminium and polyethylene are 69 GPa and 3.5 GPa, while the density of aluminium and polyethylene is 2700 and 1050 kg/m^3^, respectively, and was also inputted on simulations. By substituting the values of Poisson's ratio, Young modulus, and density of aluminium and polyethylene into (12), it will give the *p*-wave velocity of aluminium and polyethylene as 6400 ms^−1^ and 4000 ms^−1^, respectively. Since *p*-wave can travel very fast on aluminium, therefor 10 mm thick aluminium tendon duct could possibly mask the actual velocity of wave even though it is travelling in the void. Since polyethylene will not cause acceleration to the wave propagation speed, 10 mm thick polyethylene tendon duct will not affect the visualization of tomograms.

### 4.4. Transmission of Wave in Tendon Duct

**Figure 17:**
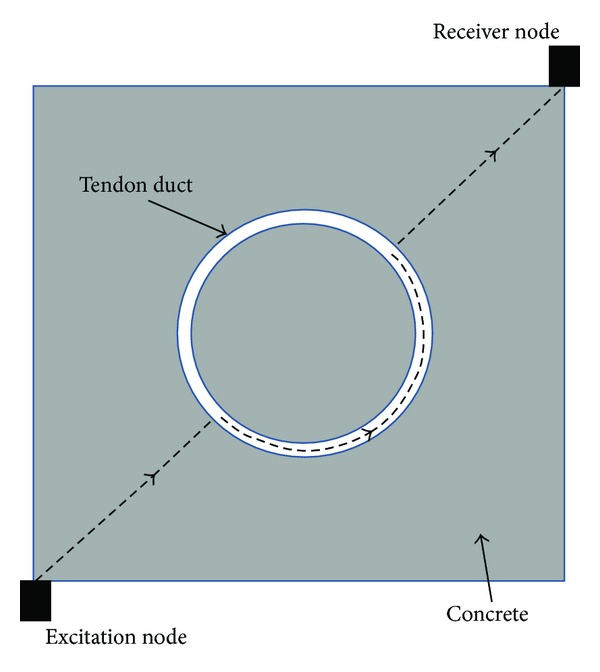
Transmission of elastic wave from source to receiver through tendon duct.

**Figure 18:**
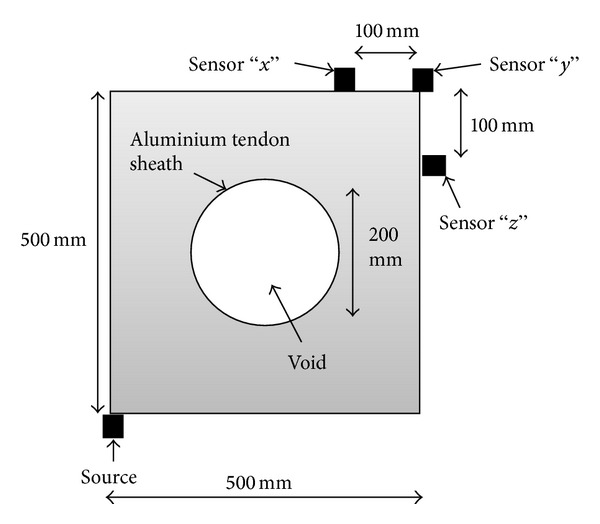
Illustration of location of sensors “*a*”, “*x*”, “*y*”, and “*z*”.

It is considered that the tendon duct can be a medium for wave to travel through. It is already known that waves propagate faster in aluminium than concrete and polyethylene. A 10 mm thick aluminium sheath is capable of acting as a pathway for the wave to travel as illustrated in Figure 17. The aluminium tendon duct now has become the shortest path for the waves to be propagated to its destination which are receiver sensors. In this condition, the receiver sensor has detected the wave travel from tendon duct instead of its actual pathway. Therefore, regardless of the filling condition inside tendon duct, visualization by tomography would not give remarkably different between grouted and ungrouted tendon duct. The set of travel times from the source to sensors *x*, *y* and *z* for 1 mm-thick and 10 mm-thick aluminium tendon sheaths with 100% void inside the tendon duct as illustrated in Figure 18 were extracted and tabulated in Table 1. It is confirmed that the wave travel time in the concrete model with 10 mm thick aluminium tendon sheath has been always faster than concrete model with 1 mm thick aluminium tendon sheath, with the difference being approximately higher than 8.5%.

## 5. Quantification of Void

**Figure 19:**
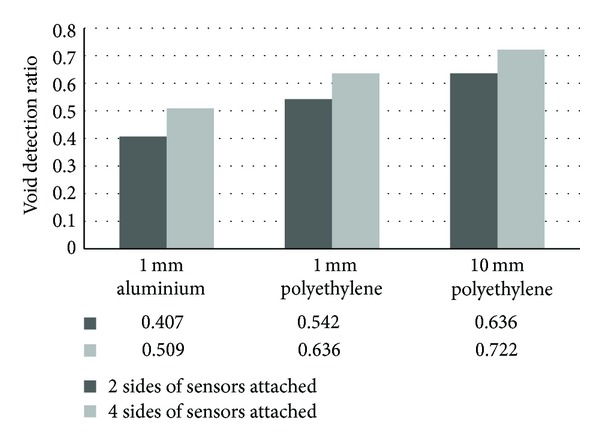
Void detection ratio for models with two-side and four-side sensor arrangements.

Figure 19 presents void quantification results of models simulated with 100% void inside tendon duct, computed as void detection ratio against the type of tendon sheath material. The ratio is calculated by proportioning the region with velocity less than 3900 ms^−1^ as indicated by tomography result with that of the actual modeled area. Three types of tendon sheaths were compared, namely, the 1 mm aluminium, 1 mm polyethylene, and 10 mm polyethylene. All the models were simulated using 100 elements. In this figure, the ratio closest to 1.0 is considered the most accurate. Based on the results, it is understood that the most effective material in visualization of void is 10 mm thick polyethylene tendon sheath followed by 1 mm thick polyethylene and 1 mm thick aluminium. It is also confirmed that the four-side sensor arrangement yielded slightly better accuracy in detection than the two-side sensor arrangement.

**Figure 20:**
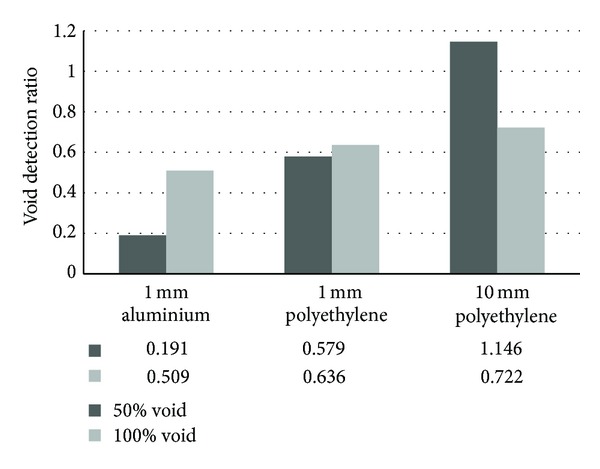
Void detection ratio for models with 50% void and 100% void.

Figure 20, on the other hand, presents the results of the void quantification comparison between models with 50% and 100% void, using different types of tendon sheaths. All the models were simulated using 100 elements. For the model with 1 mm thick aluminium tendon sheath, void detection ratio is low for both 50% and 100% voided conditions. Also, quantification for 100% void is more accurate than that of 50% void. For model with 1 mm thick polyethylene sheath, detection accuracy for both void conditions is almost similar, which is approximately 0.6. For the case of 10 mm thick polyethylene sheath, quantification accuracy is slightly in excess with ratio of 1.146 for 50% void, compared to 100% void condition, which is lower at 0.7.

**Figure 21:**
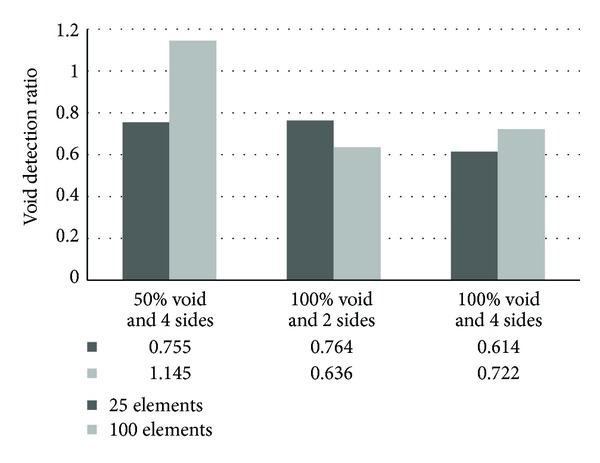
Ratio of detected void to actual void regarding the 25 elements and 100 elements.

The results of quantifying voids inside 10 mm thick polyethylene tendon sheath with different filling percentages and sensor arrangements are given in Figure 21. The result shows that for the model with 50% void inside 10 mm polyethylene tendon sheath and four-side sensor arrangement, the detection ratio is 0.8 by using 25 elements, which shifts to approximately 1.15 when the number of elements was increased to 100. This indicates that although detection was successful, estimation of the void area has been slightly excessive. Moreover, for the model with 10 mm polyethylene tendon sheath and two-side sensor attachment, better accuracy in size estimation was achieved with only 25 elements compared to using the 100 elements. While for the model of concrete with 10 mm polyethylene and four-side sensors arrangement, the quantification of void becomes better with increasing the number of element to 100. Based on the results presented by the three cases, it can be confirmed that void detection and quantification by tomography reconstruction gives a minimum of 60% accuracy, which can be considered as satisfactory.

## 6. Conclusions

Based on the study, conclusions can be made as follows.

- The visualization by tomography reconstruction technique becomes better with increasing number of sensors. Also placement of sensors on all four sides of the concrete model improves the visualization significantly. But, it is almost impossible to conduct completely coverage tomography reconstruction technique on concrete structure, so it is important to know how to interpret the tomogram with two sides of transducers coverage.
- Reducing the element/mesh size or increasing number of elements/meshing did not necessarily improve the visualization for void in tendon duct. In some cases, it led to confusion in results interpretation.
- The material type and thickness of the sheath have an influence on accuracy of visualization, because of differences in the acoustic impedance and elastic properties. This is demonstrated by the visualization for 10 mm thick aluminium tendon sheath, which is less accurate as compared with the 10 mm thick polyethylene.
- It is important to conduct preliminary studies such as numerical simulations on target structure before conducting the actual NDT on concrete structure. This is because the numerical simulations are considered to be the early design stages of NDT to check whether the NDT technique is suitable to conduct to target structure.

## Figures and Tables

**Table 1:** Set of travel time from source to sensors at locations “*x*”, “*y*”, and “*z*”.

Distance (mm)	Travel time (ms)		Percentage different between 1 mm and 10 mm thick aluminium (%)
	With 1 mm thick aluminium	With 10 mm thick aluminium	
640.312 (source to sensor *x*)	0.153	0.141	8.511
707.107 (source to sensor *y*)	0.173	0.159	8.805
640.312 (source to sensor *z*)	0.153	0.141	8.511
